# Constructing a toolkit to evaluate quality of state and local administrative data

**DOI:** 10.23889/ijpds.v4i1.937

**Published:** 2019-01-31

**Authors:** Zachary H Seeskin, Gabriel Ugarte, A Rupa Datta

**Affiliations:** 1 NORC at the University of Chicago, 55 E. Monroe Street, 31st Floor, Chicago, IL, 60603

## Abstract

In the United States, state and local agencies administering government assistance programs have in their administrative data a powerful resource for policy analysis to inform evaluation and guide improvement of their programs. Understanding different aspects of their administrative data quality is critical for agencies to conduct such analyses and to improve their data for future use. However, state and local agencies often lack the resources and training for staff to conduct rigorous evaluations of data quality. We describe our efforts in developing tools that can be used to assess data quality as well as the challenges encountered in constructing these tools. The toolkit focuses on critical dimensions of quality for analyzing an administrative dataset, including checks on data accuracy, the completeness of the records, and the comparability of the data over time and among subgroups of interest. State and local administrative databases often include a longitudinal component which our toolkit also aims to exploit to help evaluate data quality. In addition, we incorporate data visualization to draw attention to sets of records or variables that contain outliers or for which quality may be a concern. While we seek to develop general tools for common data quality analyses, most administrative datasets have particularities that can benefit from a customized analysis building on our toolkit.

## Introduction

In the United States, data held at state and local agencies for the purposes of administering programs for public assistance contain valuable information to inform policy research. Increasing the use of administrative data for statistical production and research has been identified as a priority by the Committee on National Statistics [1, 2], the Commission on Evidence-Based Policymaking [3], and the recent position statement on population data science [4]. Many public assistance programs in the U.S. are administered by state and local agencies rather than federal agencies, including the Supplemental Nutrition Assistance Program, Temporary Assistance for Needy Families, Medicaid, Unemployment Insurance, and child care subsidies. A substantial body of research shows that data from the administration of these programs provides novel measures that can inform policy and provide more accurate information on program receipt or benefit amount than data collected through surveys [5, 6, 7].

Making full use of this data requires a nuanced understanding of data quality. Extensive research has been conducted at federal statistical agencies in the U.S. and internationally regarding developing data quality framework. However, these frameworks are not necessarily suited to guide those working with data from state and local agencies, which often have fewer resources and less statistical expertise than federal statistical agencies [8]. The literature offers little guidance to staff and researchers working with state and local administrative data as to how to adapt these frameworks to evaluate such data sources’ suitability for policy research.

As documented by Allard et al. [8], there is great variation in the sophistication of different state and local agencies to assess the quality of their administrative data, with many encountering great difficulties. IT systems may be outdated and may not support analysis of performance and outcomes. Budget cuts over the past decade have led to resource constraints and limited staff availability to manage such data. Data entry errors, missing data, and duplicate records are common, harming inferences when using the data for analysis. Best practices and mature tools are needed to guide analysts working with such data sources. Further, many agencies are partnering with academic researchers working with administrative data, and these researchers may need guidance to the issues common to administrative datasets.

We construct a toolkit providing a data user with programs to readily analyze quality of their data source for conducting research on a policy question of interest, based on best practices for assessing state and local administrative data. We include components to help assess data accuracy and check the completeness of the data. Additionally, as many research questions involve comparisons among groups, among geographies, or over time, understanding possible risks to comparability is critical to inform possible caveats for analysis. The toolkit is designed in the context of the U.S., for which many federally funded programs are implemented by states, which develop their own policies as long as federal requirements are met.

We incorporate descriptive statistics and rich multivariate visualization upon finding the value of these methods for detecting possible data quality issues requiring further investigation. Our implementation uses R Markdown so that a user can readily see how code can be adapted to suit their needs and to present results in reports.

Mirroring the situation of most state agencies, the toolkit analyzes a dataset on its own, and so does not include tools to assess the data by comparing it to values from another source or dataset. It is possible, however, to compare multiple geographic units within a dataset if the data contain multiple states or cities. While some analyses are challenging in the absence of separate validation data, such as assessing potential over- and undercoverage of the dataset, we focus on checks from the literature that can analyze a data source on its own. This results in sometimes indirect measures of quality, while helping identify irregularities so that analysts can handle them knowingly.

The rest of this article proceeds as follows. First, we review the literature for assessing administrative data quality based on the work of statistical agencies in the U.S. and internationally that informs what the toolkit includes. Then, we describe the data quality toolkit and its main features. We discuss the challenges encountered in developing this toolkit including limitations of what can be included before concluding and providing recommendations for future efforts to support the usability of state and local administrative data for policy analysis.

## Background

Data quality depends on “fitness for use,” that is, whether the data meet the requirements for a specific need. An assessment of data quality is, therefore, always embedded in a decision- theoretic context, and according to Karr et al. [9], “[depends] on the capability of data to be used effectively, economically and rapidly to inform and evaluate decisions.”

A unified data quality framework helps researchers working with new data to organize their assessment for the use of interest. Several quality frameworks have been developed by international statistical agencies to judge administrative data quality. This includes frameworks built by Nordic countries building on their considerable experience with using administrative data [10, 11]. It also includes joint efforts among European counties to develop new methods and frameworks [12] as well as new knowledge advanced in the United Kingdom and United States [13, 14].

These frameworks conceptualize data quality as a multidimensional concept reflecting the different aspects needed in a data source to support its use to inform statistical inferences and analysis. Often cited dimensions across the literature include relevance, accuracy, completeness, timeliness, accessibility, clarity/interpretability, coherence/consistency, and comparability [15]. A description of these dimensions can be found in Table 1.

*Relevance* of the data derives from an analysis of metadata and is a crucial first step of data quality analysis. Some factors include indicators such as the suitability of the source for the research question in terms of the statistical population, units, and variables [10, 11].

*Completeness* focuses predominantly on coverage issues or, in the context of administrative data, whether the set of records of interest to study are those included in the data. It also includes when there are missing values for units in the dataset.

*Accuracy* focuses on the identification of errors, such as correctness of the units and validity of the data values. Daas et al. [12] provide more detail on indicators for the accuracy and completeness dimensions.

*Consistency*, *coherence*, and *comparability* of the data are critical when making comparisons between groups or over time. These dimensions focus on the agreement with other sources of data, as well as the uniformity of the collection and management for data from different groups and time periods. Further background is provided in a framework from the World Health Organization [16].

State and local agencies may lack detailed metadata to describe the units and variables, making analysis of relevance challenging. In addition, state-administered public assistance programs have eligibility rule changes with some frequency, which affects the assessment of other dimensions as well. Because the research questions of interest to state and local agencies often focus on comparisons of experiences and outcomes among subgroups and over time, attention to these quality dimensions before conducting research is critical for state and local data.

**Table 1: Dimensions for evaluating administrative data quality:** Note: Based on framework from Iwig et al. [14], with modifications based on other cited sources.

Dimension	Description

Relevance	The degree to which statistics meet the needs of the user, including whether the statistics produced are those needed for the use or research topic.
Accuracy	Whether data values reflect their true values and are processed correctly.
Completeness	Whether data cover the population of interest, include correct records, and do not contain duplicate or out-of-scope records. Additionally, whether units have information filled in for all appropriate fields without missing data.
Timeliness	Whether the data are available in time to inform policy matters of interest.
Accessibility	The conditions in which users can obtain and work with the data, including physical conditions and legal requirements for access.
Clarity/Interpretability	Whether data are accompanied by sufficient and appropriate metadata to understand the data and their quality.
Coherence/Consistency	Data from different sources are based on the same approaches, classifications, and methodologies, with enough metadata available to support combining information from different sources.
Comparability	The extent to which differences between statistics reflect real phenomena rather than methodological differences. Types of comparability: over time, across geographies, among domains.

## Toolkit

We designed the toolkit to help staff or researchers interested in conducting policy analysis who are new to analyzing data quality, particularly the quality of state or local administrative data. The toolkit is currently in an early stage of development, and we plan to make the first components available as open source tools from < http://www.norc.org/Research/Projects/Pages/family-self-sufficiency-data-center.aspx> in spring of 2019. The toolkit will allow a researcher to understand the quality considerations of the dataset that should inform their research plan and the interpretation of their results.

The toolkit is implemented using R Markdown, which readily enables sharing code and developing reports with result output. The scripts provide examples of data quality analyses with coding that the user can modify to the needs of their specific data sources. R Markdown is freely available to use with R and RStudio.

We designed the toolkit primarily to analyze dimensions of data quality that are important for state and local administrative data and involving quantitative analysis. Some common features of state and local data include that they: require care as they often lack clear metadata; are prepared and stored in computing systems not designed for traditional statistical datasets; may have varying quality for different variables based on their importance for program administration; represent special populations without ready official statistics available; and are subject to changes in eligibility rules over time with groups differentially affected by policy changes.

The first step for the toolkit is to provide guidance on the data format required. Toolkit analyses are designed for data in a rectangular, long format to support longitudinal analyses. Specifically, the data should be contained in one flat file where each column represents a different variable. Each row represents a record of a unit, whether it be a household, family, individual, or other entity, at a specific time point and each unit should have a row for each time period it is observed. The data should contain an identification variable and a time variable.

Recognizing that relevance has an important role in determining the degree to which the data are suitable for an analysis, the toolkit guides the user to first assess relevance to ensure that the time invested in analyzing the dataset will support the research planned. The toolkit leads the user to qualitatively assess to what degree units of analysis, the reference population, variables, timing, and domains match with the research goals.

We provide code to support analyses focusing on data accuracy, completeness of the data, and comparability of the data among groups of interest and over time, three dimensions for which quantitative measures are important and which may capture issues common to state and local data. Nonetheless, the documentation discusses other dimensions which can be analyzed qualitatively.

The toolkit is designed and written so that analyses do not need to be conducted in the order provided. Therefore, users who are interested in particular dimensions of data quality can choose a subset to run. While the toolkit is designed for longitudinal data, either cross-sectional (single time period) or time series data (no repetition of units over time periods) could also be analyzed with focus on the appropriate data quality dimensions.

The remainder of this section summarizes the long-term plan for components of the toolkit, with a summary provided in Table 2. More detailed descriptions of the plans for the initial version of the toolkit are provided in Supplementary Appendix 1. We next provide descriptions of the toolkit’s features for the accuracy, completeness, and comparability dimensions. Examples shown in this section are conducted on a simulated dataset for a hypothetical benefits program with monthly longitudinal data available over the time period 2011 to 2015 with about 1.5 million observations for 100,000 units.

### Tools for data accuracy

Accuracy includes having valid identification keys for all units and having valid variable values matching value ranges from the data documentation. The toolkit will guide a researcher to examine these issues in their data source. In the absence of a register to check units with wrongly assigned identification keys, it is still important to check that identification keys are syntactically correct. For example, the toolkit can check whether keys conform to certain syntactical structures, such as for Social Security Numbers or addresses. The toolkit also detects missing identification keys. Specifically, the toolkit guides a user to develop checks for whether their identification keys are in valid formats, with examples for common kinds of identification, and then provides tabular and graphical output that present the number and proportion of units with missing, correct, and incorrect syntax.

To check the accuracy of data values, the toolkit first guides the user to investigate whether the data values conform to the metadata before detecting possible dubious values and outliers. In addition to the metadata, there may be rules emerging from judgment or knowledge of the program that can be encoded by the user for checks. For example, persons under age sixteen should be categorised as out of the labour force for the employment variable. The tool requires the user to input all the rules and logical relations among variables and then provides tabular and graphical output for the proportion of records that pass or fail each check. Additionally, separate graphical output indicates the proportion of units with at least one failed rule, overall and for each group of interest specified. For time series and longitudinal analysis, the results are also shown for specified time intervals.

**Table 2: Summary of analyses planned for toolkit across three data quality dimensions:** 

Analysis	Description

**Accuracy**	
Validity of units	Assesses the validity of identification keys for units in the dataset
Validity of variable values	Assesses the sensibility of values of single variables and among multiple variables using the metadata
Trustworthy variable values	Determines values in data that, while valid, are suspicious from judgment or experience
**Completeness**	
Coverage of units	Assesses whether there are units that are missing or not available for the analysis
Duplicates	Looks at the occurrence of multiple registrations of identical units in the dataset
Missing values	Looks at the absence of values for the variables and analyzes item nonresponse by characteristics of interest for analysis
**Comparability**	
Distribution of variables	Assesses the distribution of relevant variables to look for incongruences with expected distributions
Relationships among variables	Looks for unexpected patterns in the relationships among variables
Time patterns	Looks for unexpected patterns in variables over time
Spell characteristics	Studies the characteristics of the spells in longitudinal analysis, such as duration and churn

The toolkit guides the researcher not only to examine whether values do not conform to the set rules, but also whether there are dubious values. The researcher can either examine values of one variable, for example households with more than fifteen members, or dubious combinations of variables, such as receipt of a means-tested benefit and high household income. In many cases, it is not possible to know whether outliers should be removed from the analysis or not. However, the user can learn about extreme values and decide to conduct a sensitivity analysis when the validity of some values is uncertain.

Distributional analysis is important for identifying possible outliers, and we find both tabular output and visualization to be valuable. As there is no single method for detecting outliers, we employ a variety of methods to give the use a rich perspective on how different outlier analyses compare. One visual approach we employ is letter-value plots, an extension of the boxplot, to provide a more detailed representation of variable distributions for the data user. Letter-value plots are more suitable to large data sets by presenting quantiles beyond quartiles, showing more information about the tails and reducing the number of extreme values identified as outliers [17].

**Figure 1: Example letter-value plots for simulated data:**
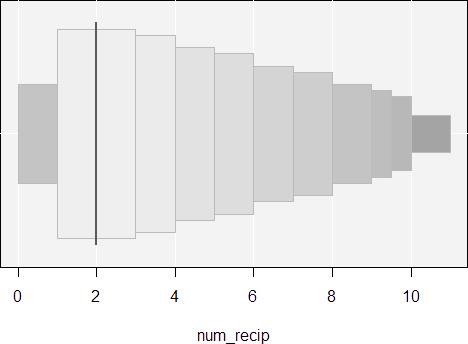

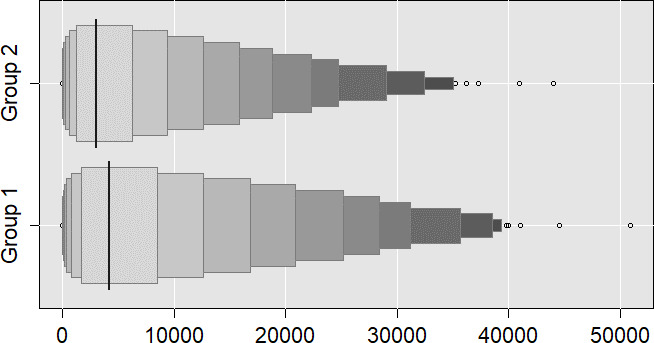
The first example is demonstrated from application of the toolkit on a simulated dataset for the variable number of recipients. Data include about 1.5 million observations over a hypothetical time period of 2011 to 2015. The second example includes simple simulations of two groups with 10,000 observations each to demonstrate the presentation of outliers. See detailed description of these figures in the text.

Examples are shown from application of the toolkit on simulated data for the variable number of recipients in Figure 1. The first example is on the simulated benefits dataset described previously with 1.5 million observations for the variable number of recipients. The second example is a simple simulation of two groups with 10,000 observations each to demonstrate the presentation of outliers. For letter-value plots, the median is presented with a vertical line, and the two boxes on either side represent the interquartile range. The next two boxes are the lower and upper eighths, while the next two are the lower and upper sixteenths. The process iterates until certain stopping rules are achieved. Any points that lie beyond the boxes are outliers that are important to investigate and are sometimes data quality issues.

### Tools for data completeness

To analyze data completeness, the toolkit provides ways to help assess both potential over- and undercoverage of the units relative to the population as well as the potential effects of item nonresponse on analysis. While assessing over- and undercoverage is challenging in the absence of a separate frame, the literature nonetheless prescribes specific checks for analyzing standalone datasets. First, to examine possible undercoverage, the toolkit guides a user to produce summaries of the number of records by geographic subareas, for example counties within a state.

This can suggest the possibility of geographic areas not included in the administrative data source or ones that are undercovered if the number of records is suspiciously low relative to population. On the other hand, duplicates are a threat to overcoverage, and the toolkit detects possible duplicates by examining repetition of the same identification keys or of a set of determined analysis variables in the dataset. This analysis can be repeated for each time point in the dataset.

We also include an item nonresponse analysis, which produces tabular and graphical representations of the absence of values for specific variables and for units with at least one missing value in any key variable. These results help to understand the impact of ignoring missing data for multivariate analysis. The toolkit also suggests the use of imputation to mitigate the effects of item nonresponse on analysis.

It is also important to characterize units with missing data in terms of characteristics that are available to all units to assess possible biases. In this case, the toolkit prescribes using potentially relevant and available complete variables to analyze missing patterns in the data. The toolkit can help the data user produce graphical output indicating when the extent of item nonresponse varies among the domains identified by the data user.

### Tools for data comparability

As many research questions involve comparisons among groups, among geographies, or over time, understanding possible risks to comparability is critical to inform possible caveats for analysis. The toolkit includes analysis of variable distributions, multivariate relationships, time series patterns, and spells, or the periods for which a unit is observed in the data.

Analyzing variable distributions is critical to detect possible irregularities when a distribution deviates from what would be expected. Often judgment is needed to determine whether the distribution indicates an issue. For example, one might expect that the distribution of the income of benefit recipients will be skewed to the left, with many units having zero income. However, jumps in the distribution of this variable to the right of zero should be carefully studied. The toolkit provides code to produce tabular and graphical output for variable distributions, including tables of quantiles and histograms with bins appropriately sized for the range of the variable.

Going beyond univariate analysis, examining irregularities in multivariate relationships is important for evaluation the fitness for use of data. Theory can help establish what relationships may be expected. For example, in some benefit programs, the cash assistance received by the family is expected to be directly proportional to the number of beneficiaries in the household. Sharp deviations from such patterns or certain jumps in the relationships can suggest potential threats to comparability.

**Figure 2: Example letter-value plots for simulated data:**
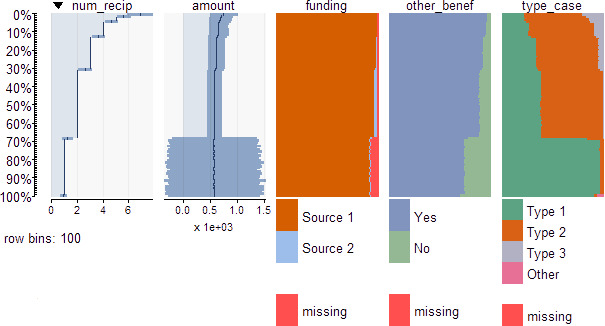
This example is demonstrated from application of the toolkit on a simulated dataset. Data include about 1.5 million observations over a hypothetical time period of 2011 to 2015. See detailed description of this example the text.

We have found using multivariate visualization to be particularly helpful for such analysis. The toolkit uses tableplots [18] to help assess relationships among two or more variables, with a demonstration in Figure 2 conducted on the previously described simulated dataset. The five variables number of recipients, benefit amount, funding source, receipt of other benefits, and type of unit or case are represented in columns. Within a variable, all records are sorted by the values of a chosen variable in the table, in this case number of recipients. The sort variable is partitioned into percentiles with its approximate empirical cumulative distribution function drawn. As a result, the y axis reflects percentiles of the distribution of the sort variable, sorted in descending order. Thus, for the dataset of about 1.5 million observations, there are around 15,000 observations in each row. All other variables in the remaining columns have their values graphed at the different values of the sort variable in the first column. For continuous variables, the black line represents the mean value at the corresponding value range of the sort variable, and the dark blue region represents values within one standard deviation of the mean. For categorical variables, different colours indicate the frequency of different categories at that value range of the sort variable. In this example, it is apparent that benefit amount has greater variability when the number of recipients is 1, a pattern that the researcher should understand before conducting data analysis. Patterns with number of recipients are also apparent for the categorical variables.

Tableplots are valuable for investigating multivariate relationships by comparing a set of variables with the sort variable, and for noting oddities such as jumps in distributions that suggest possible data quality issues. When irregularities are detected, the toolkit recommends analyzing different subgroups to assess whether that pattern emerges in specific subgroups. In many cases, it may be necessary to consult the program history and operational documents to understand whether an irregularity reflects good data or anomalies in collection or implementation practices. The user should include variables based on those of importance for their analysis. For comparisons among groups, the domain variables are recommended.

**Figure 3: Example letter-value plots for simulated data:**
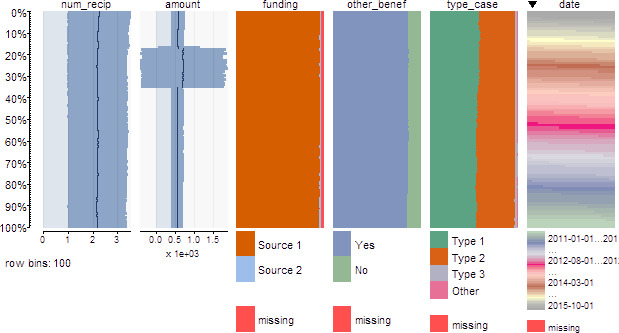
This example is demonstrated from application of the toolkit on a simulated dataset. Data include about 1.5 million observations over a hypothetical time period of 2011 to 2015. For time series analyses, tableplots are recommended with time as the sort variable (sixth column). In this example, an irregularity is detected in the second column for benefit amount.

Comparability over time is critical for time series and longitudinal analyses, and we again find that using rich visualization can help to detect and identify issues requiring further investigation. The researcher will want to investigate if there are any changes to the data collection practices or irregularities that harm comparability. First, the toolkit includes guidance to produce time series graphs of analysis variables to understand relationships over time, guidance to understand issues like seasonality, and to detect strange jumps in distributions that may reflect irregularities. Tableplots are once again provided to investigate comparability over time for many variables at once by using time as the sort variable. An example on the simulated dataset is presented in Figure 3 with time as the sort variable in the last column. For benefit amount in 2015, a change in the average and increased variability are apparent. This is a possible data quality issue that should be investigated to understand the potential impact on analyses, using methods described in the previous paragraph. The toolkit further guides users to produce tabular and graphical output describing summary statistics for subgroups of interest over time.

Further, the toolkit also includes checks that exploit the longitudinal nature common to these datasets, including that a unit has data repeated at multiple time points. The toolkit detects units with changes in variables that are expected to be stable, such as demographic characteristics, and with illogical patterns, such as decreases over time in the number of months receiving benefits.

Finally, because many programs offer a recipient benefits over a certain period time, or a spell [19], the toolkit will provide descriptive statistics for the spells, including number of spells per unit, summary statistics on spell durations, and churn, when a recipient stops receiving benefits and soon resumes them. Understanding spells is a critical component of longitudinal analyses for many research questions. For example, it is important to understand patterns in how households’ economic well-being evolves between when they begin and end receiving benefits as well as why they may have stopped receiving benefits. As for previous dimensions, statistics can be examined by subgroups and geographic areas.

## Discussion

In the process of developing this toolkit, we encountered several challenges that were difficult to address with a general toolkit. We detail these issues here to provide guidance for interpreting output from the toolkit and to inform efforts to enhance the use of state and local administrative data sources in the future.

### Indirectness of measures of data quality

Our toolkit analyzes a standalone administrative data source’s quality without comparison of the data values to those of other data sources. While it is common in the literature to compare an administrative data set with another data source [6, 20-24], rarely is a true “gold standard” available that can provide direct measures of error. More indirect measures are commonly prescribed in the literature and provided in our toolkit, particularly when a second validation data source is not available. This is a common situation for state and local agencies, as the process for obtaining access to a second dataset and linking the administrative data source is challenging. As an example, a researcher can perform many checks to see whether a data value is dubious and incompatible with other information provided for that unit, but the fact that a value is accepted as not dubious does not mean that it is correct. When such patterns are not detectable but systematic, this can result in bias for the resulting estimates. This is not an issue solely for our toolkit, but a general challenge for the administrative data quality literature. Thus, we caution anyone conducting data quality assessment that without validation data, there are limits to what these recommended quality assessments can detect.

### Quality of metadata and documentation

For state and local administrative data, quality of the metadata and the documentation can be a pronounced challenge, as providing the detailed documentation usually expected for statistical analysis of such data sources can be a burden on these small agencies. It is also more difficult for statistical agencies and researchers to provide feedback to these agencies as compared to federal programs managed by a single agency. Even for centralized datasets, there are situations where the data contained in certain fields may not contain the variable intended or reflected. As an example from an older data source, Aaronson and Mazumder [25] describes that for 1943 Army General Classification Test data, test score data were found in the weight field. A toolkit alone cannot overcome these kinds of issues. Rather, in our toolkit, we provide language to guide a data user to gain as full an understanding of the documentation as possible and to understand the limits that poor documentation can place on the ability to conduct policy research.

### Importance of understanding legal and programmatic changes

Administrative data from federal, state, and local programs are affected by changes in policy and in law. This creates a potential threat to comparability of the data over time, as a legal change may result in changes in what is collected and measured in the dataset. For example, when there are major changes to the tax code, the data collected on tax forms can change and may impact time series estimates constructed from income tax records [26]. As a second example, the recent adoption of the Community Eligibility Provision for subsidized meals in school, whereby entire schools can provide free lunches to all students if a certain percentage of students are below a poverty threshold, has impacted research using student receipt of free or reduced-price lunch as a measure of poverty [27, 28]. While we provide language to guide the user to study the background of these programs and understand any policy changes that may affect comparability, the toolkit alone cannot fully solve this issue or provide direct guidance on how to conduct analysis for some specific policy changes that may arise. Referring to the program history and operational documents is recommended to understand important legal and programmatic changes.

### Limits to generalizability

Finally, any toolkit designed to be general and apply to a range of datasets cannot resolve all quality issues encountered in different state and local administrative datasets. Each dataset has its own particularities in how it is structured or different kinds of research questions it can support, and a toolkit alone will not be sufficient for a full data quality analysis of a dataset. Rather, the toolkit makes accessible some best practices for data quality assessment for issues state and local data users commonly encounter. It provides a basis for data quality analysis and encourages the data user to understand the full context of their data source. A researcher should go beyond the toolkit to determine whether the data suit their research needs.

## Conclusions

The proposed toolkit reflects best practices from the literature on administrative data quality to help researchers working with data from state and local agencies navigate the quality issues that can arise to inform their policy research. We focus on the U.S. context, for which many federally funded programs are implemented by states constructing different policies within the federal requirements. Because these data are so promising for research, yet state and local agencies have limited budget and staff time for conducting data quality assessment, we provide a much-needed resource to make assessment of these sources possible. While statistical agencies have produced more general data quality frameworks, we disseminate specific code for data quality analyses via R Markdown to allow ready analysis and statistical reporting on data quality. The toolkit assists users with assessing data accuracy, completeness, and comparability among groups or over time. It provides direction on how to assess data quality in the context of cross-sectional, time series, or longitudinal analyses. It also identifies possible data issues that should be further investigated and provides guidance on next steps when a possible issue is detected.

This effort is still in an early stage and may inform the construction of other related tools in the future. Our focus is on analyzing a single administrative dataset from a state or local agency as a standalone source. However, data quality analysis can be enriched when there is a second data source that can be used to validate the data—either for individual records through record linkage or by examining estimates in aggregate when the same reference population can be studied in subsets of both data sources. We believe a similar toolkit designed to use a second data source for validation would be valuable as well.

Some issues with administrative data are hard to overcome with a toolkit that aims to be generalizable, including the indirectness of many data quality measures, assessing the quality of the metadata and documentation, the influence of legal and programmatic changes on the data, and the fact that any dataset has particularities that cannot be addressed with a general tool. We address these concerns in part in our documentation and emphasize the importance of going beyond the toolkit to conduct a full data quality analysis. Nevertheless, our toolkit can provide a solid basis for analyzing quality of administrative data and fulfills a need that has not been previously met.

## Supplementary Appendix 1

**Components of Toolkit Planned for Initial Version:** Notes: Each analysis can be conducted for a specified time range of interest in the data. Initial version of toolkit expected to be made available open source in spring of 2019.

Component	Description	Coding Steps	Report Output

**A. File Setup**			

1. File setup	Specifies format of file needed for use in toolkit, specifically rectangular, long format. This section needs to be set up prior to running the report.	Reads in file and makes important specifications, including ID variable, time variable, and other variables for analysis.	Provides written language for important requirements for file setup.
**B. Relevance**			

2. Assessing relevance	Describes aspects needed to assess relevance, including units, variables, and population.	No coding required.	Directs user to review metadata and program history.
**C. Accuracy and Completeness**			

3. Technical checks	Conducts checks of whether data values are sensible and conform to the codebook.	Runs checks for invalid values of variables. Can be based on user input specified by user.	Provides table with counts of invalid values for each variable, with direction for setup and description of output.
4. Descriptive statistics for numeric values	Provides tabular output on variable distributions including quantiles, as well as extent of item missing data for a specified time range.	Runs statistics for all variables formatted as numeric.	Presents tables and describes the kinds of anomalies user should examine.
5. Descriptive statistics for character Values	Provides tabular frequencies for values as well as extent of missing data.	Runs statistics for all variables formatted as categorical.	Presents tables and describes the kinds of anomalies user should examine.
6. Distributions, extreme values, and outliers	Provides tabular and graphical output to determine outliers, including by applying Tukey fences, boxplots, and letter-value plots.	Runs statistics and graphs for all variables formatted as numeric, with graphical options adjusting based on variable format.	Presents series of tables and graphs and describes how to interpret each, including using aggregate findings to detect potential issues.
7. Examining missing data patterns	Provides bar plots of item nonresponse by domain variables.	Runs item nonresponse statistics and graphs by specified domain variables.	Presents item nonresponse graphs and provides interpretation including suggestions for mitigating effects of nonresponse on analyses, including imputation.
**D. Comparability**			

8. Examining variable distributions	Provides histograms or bar plots of distributions as appropriate, and guides user to assess whether distribution has irregularities.	Runs graphs for analysis variables choosing correct graph based on format of data.	Presents histograms and bar plots discussing interpreting each in terms of domain knowledge regarding expected distributions, in context of threats to comparability.
9. Assessing patterns in relationships among variables	Uses tableplots to assess irregular patterns in relationships among analysis variables.	Runs tableplots of designated analysis variables, one set sorted by each analysis variable.	Presents tableplots and provides detailed description of how to interpret and kinds of patterns suggesting anomalies.
10. Assessing patterns over time	Uses tableplots sorted by time to assess comparability of data over time.	Runs tableplots of analysis variables sorted by time.	Presents tableplots and provides detailed description of how to interpret, specifically kinds of patterns suggesting anomalies.
